# Management of vesicovaginal fistula: An experience of 52 cases with a rationalized algorithm for choosing the transvaginal or transabdominal approach

**DOI:** 10.4103/0970-1591.36709

**Published:** 2007

**Authors:** Rakesh Kapoor, M. S. Ansari, Pratipal Singh, Parag Gupta, Naval Khurana, Anil Mandhani, Deepak Dubey, Aneesh Srivastava, Anant Kumar

**Affiliations:** Department of Urology and Renal Transplantation, Sanjay Gandhi Post Graduate Institute of Medical Sciences, Lucknow, India

**Keywords:** Fistula, management, tuberculosis, vesicovaginal

## Abstract

**Purpose:**

We aim to present our experience for the repair of vesicovaginal fistula (VVF) with special reference to surgical approach.

**Materials and Methods:**

From January 1999 to June 2005, 52 VVF patients with mean age of 32 years underwent operative treatment. Fistulas were divided into two groups, simple and complex, depending on site, size, etiology and associated anomalies. Simple VVFs were approached through the vaginal route and complex VVFs via the transabdominal route. Patients were evaluated at two to three weeks initially, three-monthly twice and later depending on symptoms.

**Results:**

Thirty-two (61.5%) had simple fistulas and 20 (38.5%) complex fistulas. The most common etiology was obstetric trauma in 31 (59.6%) patients, while the second most common cause was post hysterectomy VVF. Thirty-two (61.5%) patients were managed by transvaginal route, of which 17 had supratrigonal and 15 trigonal fistulas. Twenty (38.5%) patients with complex fistulas were managed by abdominal route. The mean blood loss, postoperative pain and mean hospital stay were shorter in transvaginal repair. Eleven (21.2%) patients required ancillary procedures for various other associated anomalies at the time of fistula repair. Three patients failed repair giving a success rate of 94.2%. At a mean follow-up of three years 48 women were sexually active, of these 10 (19.2%) complained of mild to moderate dyspareunia.

**Conclusion:**

Most of the simple fistulas irrespective their locations are easily accessible transvaginally while in complex fistulas we recommend the transabdominal approach. Depending on the clinical context both the approaches achieved comparable success rates.

Vesicovaginal fistula (VVF), commonly caused by prolonged obstructed labor, is one of the worst complications of childbirth and poor obstetric care in the developing world. This unpleasant complication leaves affected women with continuously leaking urine, excoriation of vulvas and vaginas, often rendering them social outcasts.[1–3] The key to successful repair of VVF lies in the classic principles defined by Couvelaire in 1953, “good visualization, good dissection, good approximation of the margins and good urine drainage.[4] These principles can be achieved both through vaginal and abdominal approaches.

Although the choice of technique partly depends on the characteristics of the fistula (site, size, clinical context), it also largely depends on the experience of the surgical team. Most of the simple VVFs both trigonal and supratrigonal, can be easily managed thorough the transvaginal route, specially by using simple maneuvers to bring the fistula closer to the operating surgeon.[5,6] More complex fistula requires transabdominal route for optimal repair.[7–9] Herein we describe our approach in managing patients both with simple and complex VVFs and formulate an algorithm to choose the best approach based on our experience.

## MATERIALS AND METHODS

From January 1999 to June 2005, 52 patients with mean age of 32 years (range 17 to 53 years) with vesicovaginal fistulas underwent surgical repair at our institution. These included all patients presenting to us during this period and were referred to us by primary and secondary healthcare centers as well as by private practitioners. This is a retrospective study and data was recorded on etiology, site, size and numbers of fistulas; surgical approach and ancillary procedures required; complications and their sexual rehabilitation. All patients were evaluated preoperatively by history, physical examination, serum creatinine, ultrasonography abdomen and intravenous urography (IVU). Cystoscopy was performed to determine the site, size and numbers of the fistulas along with the assessment of the mucosa around the fistulous opening. Vaginal speculum examination was done to asses the vaginal capacity and vaginal mucosal integrity. On the basis of site, size, etiology and associated anomaly, fistulas were divided in two groups, simple and complex. Primary fistula greater than 4 cm in size or recurrent fistula greater than 2cm in size, fistula involving urethra and/or bladder neck, fistula requiring ureteric reimplantation/augmentation cystoplasty, fistula with large bladder stone or fistula with scarred and non capacious vagina and *post radiotherapy fistula* were considered as complex fistulas while the rest as simple fistula. Fistula repair was done through either the vaginal or abdominal route, which was decided by the type of fistula i.e. simple or complex. Primary fistulas were repaired once local vaginal tissue was healthy and infection-free while for recurrent or obstetric fistulas repair was delayed for at least three months or unless infection-free. Simple VVFs were approached through the vaginal route and complex VVFs via the transabdominal route.

### Vaginal repair

**Figure 1:**
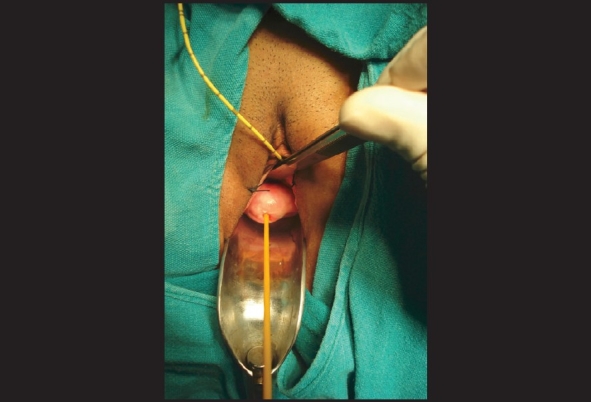
Traction on a small-sized Foley catheter, which is passed into the bladder through the fistulous opening, helps to bring the fistula closer to the view

**Figure 2:**
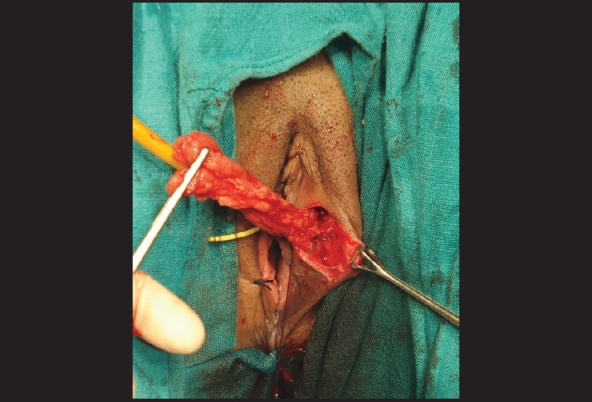
Martius flap raised, which was used as interpositional flap in transvaginal repair

When the vaginal route was used and ureteric orifices were close to the fistula, both the ureters were catheterized cystoscopically to safeguard the ureters. After identification of fistula, a small-sized Foley catheter was passed through it in the bladder directly or after minimal dilatation of fistula tract in case of small fistula and balloon was inflated. Traction on Foley catheter helped to bring the fistula closer to the operating surgeon [Figure 1]. When the fistula was very small, a guide wire was passed through it and fistula hole was dilated so as to pass a small Foley catheter over the guide wire. Vaginal mucosal ‘U’-shaped incision was given after saline infiltration into the mucosa. A generous plane between the bladder and vagina was developed at least 2 cm. beyond the fistulous opening to get adequate vaginal flaps for layer-wise closure. Fistula was closed without excising it and its walls were included in the first layer of closure which provided a strong anchor of supporting tissue. The fistula repair was done in three layers. First layer was created by approximating the fistula edges at the bladder wall. Second layer was created by approximation of perivesical fascia over the first layer. Third layer of repair involved the closure of vaginal flaps. Interposition graft was taken from labial fat or peritoneum of cul-de-sac in all patients [Figure 2]. Bladder was drained with per urethral catheter only in all patients for 14 days postoperatively.

### Abdominal repair

Fistula repair through the abdominal route was done using the O'Connor technique.[8] Cystoscopy and ureteric catheterization was done before opening the abdomen. Bladder was bivalved till the fistula site and then was dissected off the vagina. Bladder and vagina were closed separately and interposition graft was placed in all using omentum or pelvic peritoneum. Suprapubic and per urethral catheter was placed for bladder drainage for 14 days postoperatively.

All patients were kept on anticholinergics to avoid bladder spasms and full-dose antibiotics in the perioperative period for three days and then discharged on 100 mg ciprofloxacin daily till the catheter was removed.

Patients were evaluated at two to three weeks initially and three-monthly later twice and later on depending on the presence of symptoms. Abstinence from sexual intercourse was advised for three months postoperatively. Sexually active were defined as women participating in vaginal intercourse for this study and data were recorded in case files.

## RESULTS

The demographic profile of all patients is given in Table 1. Thirty-two (61.5%) patients had simple fistulas while 20 (38.5%) had complex fistulas. The locations of these fistulas were supratrigonal (18), trigonal (24), mixed trigonal and supratrigonal (10). Fifteen (28.8%) had recurrent fistula with history of only one previous surgery. The most common etiology was obstetric trauma i.e. 31 (59.6%) out of 52 patients, while the second most common cause was post hysterectomy VVF [Table 1]. Thirty-two (61.5%) patients were managed by the transvaginal route, of which 17 had supratrigonal and 15 trigonal fistulas. Trigonal fistulas (three) up to 4 cm and supratrigonal fistulas (one) < 3 cm in size could be accessed through the vaginal route. Twenty (38.5%) patients with complex fistulas were managed through the abdominal route [Table 2]. The sites of fistulas in these patients were trigonal (nine), supratrigonal (one) and trigonal plus supratrigonal (10). Reasons for transabdominal repair were: poor vaginal capacity (three), large fistula size (>4 cm) and closeness to ureteric orifice (six), extensive fibrosis around fistula with nonpliable vagina (five), augmentation cystoplasty (three), open cystolithotomy (two) and non-visualization of ureteric orifice on cystoscopy in one patient. Routes of fistula repairs according to their etiology are given in Table 3. While comparing the two approaches we found lesser amount of mean blood loss (250 vs. 400 ml), shorter mean operative time (98 vs. 167 min) and shorter mean hospital stay (seven vs. 10 days) in transvaginal repair as compared to transabdominal repair. Transvaginal repair was also associated with decreased requirement of analgesics (mean Tramadol hydrochloride 215 mg vs. 438 mg).

**Table 1:** Demographic profile of patients

Variables	No of patients (n= 52)
Mean age (years)	32 (range 17-53)
Type of fistula	
Primary	37
Recurrent	15
Nature of fistula	
Simple fistula	32
Complex fistula	20
Location of fistula	
Trigonal	24
Supratrigonal	18
Mixed	10
Etiology	
Obstetric trauma	31
Post Hysterectomy	15
Bladder stone	03
Genitourinary tuberculosis	02
Iatrogenic	01

**Table 2:** Characteristics of complex fistula

Fistula greater than 4 cm	07
Recurrent fistula greater than 2 cm	03
Fistula requiring ureteric reimplantation	06
Fistula requiring bladder augmentation	03
Fistula involving urethra	02
Fistula due to genitourinary tuberculosis	02

**Table 3:** Routes of fistula repair according to etiology of fistula

Etiology of fistula (including recurrent fistula)	Vaginal repair (n=32)	Abdominal repair (n=20)
Obstetric (n=31)	21	10
Post hysterectomy (n=15)	10	5
Bladder stone (n=3)	0	3
Genitourinary tuberculosis (n=2)	0	2
Iatrogenic (n=1)	1	0

We used interposition flaps in all but one. In transvaginal repair we used Martius flap (30) and peritoneal flap (one) as interposition tissue while in abdominal repair it was omentum (16), peritoneum (three) and both omentum and peritoneum (one).

Eleven (21.1%) patients required ancillary procedures for various other associated anomalies at the time of fistula repair. These included percutaneous cystolithotomy, total abdominal hysterectomy with left salpingo-oophorectomy, coloanal anastomosis with right ureteric reimplantation with bladder neck closure with Mitrofanoff, right nephroureterectomy with Struder pouch with left ureteric reimplant, urethrovaginal fistula repair, sigma rectal pouch and mesh hernia repair in one patient each while open cystolithotomy and augmentation cystoplasty with hysterectomy with bilateral ureteric reimplantation in two patients each.

Among the patients with recurrent fistula, 10 patients had primary surgery by vaginal route while five had by vaginal route. These were repaired irrespective of primary route used but depending on the fistula size and condition of vagina. Twelve were treated via the vaginal route while three via the abdominal route and all had successful outcome.

No patient undergoing only VVF repair in the present series had urinary incontinence. However, patients with urethrovaginal fistula repair had mild stress urinary incontinence which was managed conservatively while patients who required augmentation cystoplasty (n=2) or orthotopic neobladder (n=1) required clean intermittent catheterization to completely empty the bladder.

We had three failures with a success rate of 94.2%. Of these three patients, two had initial surgery by abdominal route while one had through vaginal approach. Recurrent fistula size was less than 1 cm in two patients, which were managed by the transvaginal approach while the third patient required ileal conduit as the bladder was too small and affected with genitourinary tuberculosis along with nonpliable scarred vagina. At a mean follow-up of three years (range five months - 5.5 years) 48 women were sexually active and of these 10 (19.2%) complained of mild to moderate dyspareunia, the intensity of which gradually reduced over one year since the time of fistula repair.

## DISCUSSION

Vesicovaginal fistulas are among the most distressing complications of obstetric and gynecologic procedures. The condition is a socially debilitating problem with important medicolegal implications. In contrast to the western world, obstetric VVFs remain a major medical problem in many underdeveloped countries with a low standard of antenatal and obstetric care.[9–11] In contrast to the postsurgical fistula, which is usually the result of more direct and localized trauma to otherwise healthy tissues, the obstetric fistula is the result of a “field injury” to a broad area that results in wider area of damage; thus producing a larger size of fistula.[12] Various methods of fistula repair have been described, Latzko procedure, open transabdominal, transvaginal, laparoscopic, transurethral endoscopic and urinary diversion depending on the characteristics of the fistula.[11,13,14] The vaginal approach essentially involves adequate exposure and dissection of fistulous tract along with layered closure of the fistula with or without an interpositional flap.[15–17] The most frequently used abdominal approach nowadays is the O'Connors bivalve technique.[8] The success rate has varied between 75–95% with these various techniques. [11–18]

In spite of the management being better defined and standardized over the last decade the surgical approach has always been an issue of contention for the repair of VVF. The fundamental treatment principles for VVF repair (adequate exposure, tension-free approximation of the fistula edges, nonm overlapping suture lines, good hemostasis, watertight closure and adequate postoperative bladder drainage) can be achieved through both, vaginal and abdominal route, depending upon the surgical experience. Transvaginal exposure of vesicovaginal fistulas may be a little challenging but it has been shown to be associated with less blood loss, morbidity and shorter hospital stay.[6,12]

The factors like fistula size, closeness of fistula to the ureteric orifice and time interval of injury now hardly affect the choice of repair and nowadays there is a trend more towards the transvaginal approach.[12,17] Transvaginal exposure of VVF may be a little difficult which may be lessened by catheterization of the fistula with a Foley catheter and use of the inflated balloon for traction enables the operating surgeon to pull it closer to view.[5,6,12] We too found this maneuver quite useful in most of our patients with both trigonal as well as supratrigonal fistulas. We did not excise the fistulous tract or the involved vaginal cuff for fear of enlarging the fistula size. Moreover, raising adequate vaginal and bladder flaps obviates the need of these two steps.[12,17]

In the present study 32 patients were managed transvaginally out of which 17 had supratrigonal fistulas and these were the patients in whom traction by catheter placed through the fistula helped us in bringing the fistula closer to view thus making the vaginal approach quite convenient. Even recurrent fistulas up to 2 cm (n=12), which had occurred after prior failed repair done elsewhere could also be done through the vaginal route. However, when the fistula is complex vaginal exposure of the fistula is suboptimal which may compromise the repair or endanger the ureters. In these circumstances, a transabdominal approach should be considered. We had 20 cases of complex VVFs, which were managed with the same technique.

Obstetric trauma (59.6%) remains the predominant cause in our cases that gave rise to a wider fistula secondary to field injury effect which is comparable to previously reported series.[12] However, the majority of post-hysterectomy fistulas in the present series are less than 2 cm in diameter which is again comparable to other previously reported series.[12,16]

Patients with small bladders need augmentation cystoplasty in addition to the VVF repair. We had three such patients, in two of these the bladder was extensively scarred, half of which had to be excised and this was managed with an ileal cystoplasty with an antireflux ileal nipple valve into which the ureters were reimplanted while the third patient opted for sigma rectal pouch.

Furthermore, to improve the results of fistula repair various grafts and flaps have been interposed between the bladder and vagina to promote healing and decrease the incidence of fistula recurrence.[10–14] Since most of our patients had poor nutritional status, previously failed repair done elsewhere and complex fistulas, we used interposition flaps in all but one. Martius labial pad of fat was the flap of choice in vaginal repair, while in the abdominal route it was omentum, if omentum was not available peritoneum was used. When compared, the two approaches i.e. transabdominal versus transvaginal, the results were quiet comparable. We found that the transvaginal approach is less morbid with less postoperative pain, early recovery and shorter hospital stay.

Recently, the laparoscopic approach has been used for VVF repair which follows the same principles as of standard abdominal approach, however, only limited numbers of patients are reported till date. The largest reported series comprises 15 cases with mean operative time of 2.8h, mean hospital stay of three days and success rate of 93% at mean follow-up of 26.2 months.[18] However, larger series are required to establish this approach and the cost-effectiveness of this procedure remains an issue considering that VVF is a disease of developing countries.

**Figure 3:**
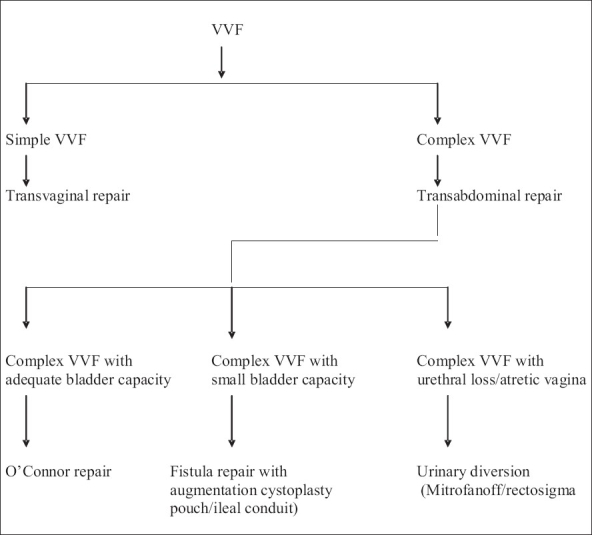
Algorithm for the treatment of VVF

Because of the retrospective nature of our analysis, we recognize its limitations. The two groups were not statistically comparable therefore differences among the groups were not compared. The surgical approach was not randomized and the decision to use an interposition flap was solely based on the reasons mentioned above. However, we propose a simple algorithm for the management of these patients based on our experience [Figure 3].

## CONCLUSION

The approach for the management of VVF has to be individualized depending on the local findings. Most of the simple fistulas irrespective of their locations are easily accessible transvaginally. The transvaginal approach is less invasive and achieves comparable success rates. We recommend transabdominal approach for complex fistula, which allows simultaneous correction of associated anomaly. It is acceptable to repeat the repair through a vaginal approach even after a first vaginal or transabdominal failure or vice versa.

## References

[1] Latzko W (1942). Postoperative vesicovaginal fistulas: Genesis and therapy. Am J Surg.

[2] Zimmern PE, Hadley HR, Staskin DR, Raz S (1985). Genitourinary fistulae. Vaginal approach for repair of vesico-vaginal fistulae. Urol Clin North Am.

[3] Carr LK, Webster GD (1996). Abdominal repair of vesicovaginal fistula. Urology.

[4] Couvelaire R (1953). Reflections on a personal statistics of 136 vesicovaginal fistulas. J Urol Medicale Chir.

[5] Woo HH, Rosario DJ, Chapple CR (1996). The treatment of vesicovaginal fistulae. Eur Urol.

[6] Stothers L, Chopra A, Raz S, Raz S (1996). Vesicovaginal fistula. Female urology.

[7] Eilber KC, Kavaler E, Rodriguez LV, Rosenblum N, Raz S (1998). Ten-year experience with vesicovaginal fistula repair using tissue interposition. J Urol.

[8] O'Connor VJ, Sokol JK, Bulkley GJ, Nanninga JB (1973). Suprapubic closure of vesicovaginal fistula. J Urol.

[9] Hadley HR (2002). Vesicovaginal fistula. Curr Urol Rep.

[10] Dalela D, Goel A, Shakhwar SN, Singh KM (2003). Vesical calculi with unrepaired vesicovaginal fistula: A clinical appraisal of an uncommon association. J Urol.

[11] Hodges AM (1999). The Mitrofanoff urinary diversion for complex vesicovaginal fistulae: Experience from Uganda. BJU Int.

[12] Arrow Smith S, Hamlin EC, Wall LL (1996). Obstructed labor injury complex: Obstetric fistula formation and the multifaceted morbidity of maternal birth trauma in the developing world. Obstet Gynecol Surv.

[13] Raz S, Bregg KJ, Nitti VW, Sussman E (1993). Transvaginal repair of vesicovaginal fistula using a peritoneal flap. J Urol.

[14] McKay HA (2004). Vesicovaginal fistula repair: Transurethral suture cystorrhaphy as a minimally invasive alternative. J Endourol.

[15] Ou CS, Huang UC, Tsuang M, Rowbotham R (2004). Laparoscopic repair of vesicovaginal fistula. J Laparoendosc Adv Surg Tech A.

[16] Sims JM (1852). On the treatment of vesicovaginal fistula. Am J Med Sci.

[17] Martius H (1928). Die operative Wiederherstellung der vollkommen fehlenden Harnrohre und des Schliessmuskels derselben. Zentralbl Gynakol.

[18] Iselin CE, Aslan P, Webster GD (1998). Transvaginal repair of vesicovaginal fistulas after hysterectomy by vaginal cuff excision. J Urol.

[19] Sotelo R, Mariano MB, Garcia-Segui A, Dubois R, Spaliviero M, Keklikian W (2005). laparoscopic repair of vesico vaginal fistula. J Urol.

